# Evaluation of HIV-DNA and inflammatory markers in HIV-infected individuals with different viral load patterns

**DOI:** 10.1186/s12879-017-2676-2

**Published:** 2017-08-22

**Authors:** Francesca Falasca, Daniele Di Carlo, Corrado De Vito, Isabella Bon, Gabriella d’Ettorre, Alessandra Fantauzzi, Ivano Mezzaroma, Caterina Fimiani, Maria Carla Re, Vincenzo Vullo, Guido Antonelli, Ombretta Turriziani

**Affiliations:** 1 Department of Molecular Medicine, “Sapienza” University of Rome, Viale dell’Università 31, 00185 Rome, Italy; 2 Department of Public Health and Infectious Diseases, “Sapienza” University of Rome, Rome, Italy; 3 Microbiology Section of the Department of Experimental, Diagnostic and Specialty Medicine, School of Medicine, University of Bologna, Bologna, Italy; 4 Department of Clinical Medicine, “Sapienza” University of Rome, Rome, Italy; 5 Umberto I University Hospital, Rome, Italy

**Keywords:** HIV, Inflammation, HIV-DNA, HIV-RNA

## Abstract

**Background:**

Persistent residual viremia (RV) and low grade inflammation and immune activation have been associated with non-AIDS defining events. The impact of persistent RV and HIV-DNA load on immune activation/inflammation remains unclear. The purpose of this study was to gain new insights into the relation between viremia, markers of inflammation and HIV-DNA levels.

**Methods:**

Three hundred and twenty-one HIV-infected patients were studied. A retrospective analysis of viremia values, prospectively collected for 48 months, was performed. Patients were separated into three groups: 113 TND (Target Not Detected, patients with sustained undetectable viremia); 113 RV (Residual Viremia, patients who had at least three detectable viral load (VL) values <37 copies/ml); 95 LLV (Low Level Viremia, patients with at least two VL values >37 but <200 copies/ml). HIV-DNA, TNF-α, IL-6 and sCD14 were analyzed.

**Results:**

HIV-DNA, sCD14 and TNF-α were significantly lower in the TND group than in the RV and LLV groups. In addition, RV patients showed lower levels of HIV-DNA and sCD14 than LLV individuals. HIV-DNA load was not related to markers of inflammation. The ordinal logistic analysis showed that two independent variables were significantly associated with VL pattern: sCD14, HIV-DNA. In addition NRTIs plus NNRTIs and NRTIs plus PIs were negatively associated to VL pattern compared to INI-containing regimen.

**Conclusions:**

Persistent undetectable viremia was associated with lower levels of inflammatory markers and HIV-DNA. However, the lack of normalization of these biomarkers in the TND group and the fact that HIV-DNA load was not associated with inflammation strongly suggest that other mechanisms play a major role in maintaining inflammation over time.

## Background

Persistent systemic inflammation has been associated with human HIV disease progression [1, 2]. Antiretroviral therapy (ART) suppresses viral load (VL), but even after long-term effective treatment HIV-infected individuals have persistent low grade inflammation and immune activation that have been associated with a number of serious non-AIDS defining events [3–7]. Persistent viral replication, residual immune dysregulation, or microbial translocation (MT) have been implicated in the mechanism underlying immune activation in these patients [8–11]. High-sensitivity C-reactive protein (hsCRP) and interleukins 6 and 10 (IL-6, IL-10) have been associated with low level HIV viremia and reported as predictors of noninfectious complications including cardiovascular disease (CVD), cancer and overall mortality [12–16].

MT can be observed in most untreated HIV-positive patients and is considered one of the main causes of chronic inflammation in HIV infection [17, 18].

Lipopolysaccharide (LPS) is considered a major marker of MT. It has been reported that circulating levels of LPS are a strong predictor of disease progression independently of viral load and CD4+ cell count [17]. LPS stimulation leads CD14+ monocytes/macrophages to secrete soluble CD14 (sCD14), which bind to LPS. Wada et al. found that sCD14 levels were significantly higher in HIV-infected compared to uninfected individuals, but did not differ between patients without or with effective ART and changed very little in the years following ART initiation [19]. Higher levels of sCD14 as well as tumor necrosis factor alpha (TNF-α), IL-6 and LPS were found in patients with MT irrespective of viremia value. However, HIV-treated patients with undetectable VL presented MT less frequently than patients with low level HIV viremia [20].

The impact of persistent residual viremia on immune activation, inflammation and microbial translocation is currently unclear. One hypothesis is that despite low viral concentrations, the ongoing presence of virus could prevent the normalization of some systemic inflammatory markers. In addition, residual viremia has been correlated with the size of the viral reservoirs as measured by HIV-DNA, indicating a link between these parameters [21–23], whereas HIV-DNA load is not associated with immune activation [24].

The purpose of this study was to further investigate the relation between the degree of residual viremia, HIV-DNA levels and inflammation. A better understanding of how these parameters are related could add information on the mechanism(s) involved in immune activation and help identify potential preventive interventions in HIV patients.

## Methods

### Study population

Three hundred and twenty-one HIV-infected patients from Umberto I University Hospital were studied. Viremia values were prospectively collected, for 48 months, as part of routine clinical practice. The analysis started at an arbitrary time point during combination ART (cART), the first viremia value quantified by kinetic PCR molecular system (see below) was considered as baseline VL. For this study we selected patients who had been HIV positive for a long period of time, showed a long time of treatment and had achieved viral suppression (HIV RNA <50 copies/ml in two consecutive VL determination), before the baseline.

Patients were grouped according to their VL pattern: group TND (Target Not Detected): patients with a sustained undetectable viremia throughout follow-up (*n* = 113); group RV (Residual Viremia): patients who had at least three detectable VL values but below the threshold value, during the study period (*n* = 113); group LLV (Low Level Viremia): patients with at least two VL values over the threshold value but below 200 copies/ml (*n* = 95). Data were analyzed at the end of the follow-up.

The study was approved by the Ethical Committee of Sapienza University Hospital (Ethical Committee authorization number # 07–09-15/3725).

### HIV-RNA evaluation

HIV-RNA was quantified using the kinetic PCR molecular system (kPCR, Versant HIV-RNA kPCR 1.0; Siemens Healthcare Diagnostics). The kPCR assay gives two possible outputs: (i) a quantitative result for HIV-RNA values ranging from ≥37 copies/mL to 11,000,000 copies/mL; (ii) a qualitative result for HIV-RNA values between 1 and 37 copies/mL (target detected) and an ‘undetectable’ result if no signal can be detected (target not detected).

### Evaluation of inflammation markers: TNF-α, IL-6 and sCD14

Levels of tumor TNF-α, IL-6 and sCD14 samples, collected and stored during the follow-up period, were measured using a commercially available enzyme-linked immunosorbent assay (ELISA) kit (Enzo Life Sciences, Inc. Farmingdale, NY, USA), according to manufacturer’s instructions. The lower limit of detection (LOD) was 6 pg/ml for IL-6, 8.4 pg/ml for TNF-α and 1 μg/ml for sCD14.

### HIV-DNA evaluation

Blood samples collected in EDTA were separated into plasma and cells by Ficoll-Hypaque density gradient centrifugation. Dry pellets were stored at −20 °C until used. Total DNA was extracted from PBMCs using a QIAamp DNA mini kit (QIAGEN, Hilden, Germany), according to the manufacturer’s instructions, to obtain 100 μl of eluate.

Total HIV-DNA was quantified using the “Generic HIV-DNA Cell” commercial kit (Biocentric, Bandol, France) according to the manufacturer’s instructions.

### Statistical analysis

The quantitative variables were expressed as the median [interquartile range (IQR)], and the categorical variables were expressed as absolute and relative frequencies.

χ^2^ test was used to evaluate percentage differences in patients between groups. Mann-Whitney with Bonferroni correction and Kruskal-Wallis tests were used to evaluate differences between CD4 cell, sCD14, inflammation markers and HIV-DNA levels among two or three groups of patients.

HIV-DNA and plasma levels of sCD14 and IL-6 were correlated by Spearman correlation test. A regression model for logistic ordinal dependent variables was built to identify factors associated with VL (TND, RV, LLV). The VL pattern was considered an ordinal dependent variable. The following variables were included in the model: gender (0 = male, 1 = female); age (years, quantitative); therapy regimen [0 = integrase inhibitors (INI), 1 = other, 2 = nucleoside reverse transcriptase inhibitors (NRTIs) + non-nucleoside reverse transcriptase inhibitors (NNRTIs), 3 = NRTIs + protease inhibitors (PIs)]; duration of therapy (years, quantitative); stage of disease (0 = A1-A3, 1 = B1-B3, 2 = C1-C3); years from testing (quantitative); HBV (0 = negative, 1 = positive); HCV (0 = negative, 1 = positive); sCD14 (quantitative); IL-6 (quantitative); TNF-α (quantitative); HIV-DNA (quantitative).

## Results

### Characteristics of the study subjects

The study analyzed 321 HIV-infected patients, 217 males and 104 females, receiving ART. The general characteristics of the patients, collected at 48 months, are summarized in Table 1. The median participant age was 53 years [IQR 47–58 years] and there was no difference among study groups in age distribution. The median CD4+ cell count was 601 cells/mm^3^ [IQR 382–854 cells/mm^3^] and median CD4+ nadir was 222 cells/mm^3^ [IQR 108–366 cells/mm^3^]. Median CD4+ cell count was slightly lower in the RV group compared to the group with undetectable viremia [565 cells/mm^3^ (IQR 302–796) vs 641 cells/mm^3^ (IQR 523–888) *p* = 0.017). Patients had been on ART for a median of 15 years (IQR 9–19 years).

**Table 1:** Study population characteristics

Characteristic	Overall *n* = 321	Group TND *n* = 113	Group RV *n* = 113	Group LLV *n* = 95	*p* ^a^
Age, y	53 [47–58]	52 [45–57]	54 [49–62]	53 [47–57]	
Male (%)	217 (67)	75 (66)	78 (69)	64 (57)	
Race/Ethnicity white/latino/other (%)	316 (98.5)	113 (100)	111 (98.2)	92 (97)	
Black (%)	5 (1.5)	0	2.0 (1.8)	3.0 (3.0)	
CD4 cells/mm^3^	601 [382–854]	641 [523–888]	565 [303–795]	565 [356–925]	*0.017*
Nadir CD4 cells/mm^3^	222 [108–366]	209 [112–362]	243 [108–327]	232 [107–409]	
HIV infection, y	16 [10–22]	16 [13–21]	15 [8.0–20]	18 [10–22]	
Duration of ART, y	15 [9.0–18.5]	15 [12–18]	13 [6.0–18]	18 [10–21]	
HCV coinfection, n (%)	55 (17.1)	14 (12.4)	24 (21.2)	17 (17.9)	
HBV coinfection, n (%)	17 (5.3)	6.0 (5.3)	4.0 (3.5)	7.0 (7.3)	

Data are median [interquartile range] unless otherwise stated

^a^Kruskal-Wallis tests

Abbreviations: *TND* target not detected, *RV* residual viremia, *LLV* low level viremia, *ART* antiretroviral therapy, *HCV* hepatitis C virus, *HBV* hepatitis B virus

### Markers of systemic inflammation

**Fig. 1:**
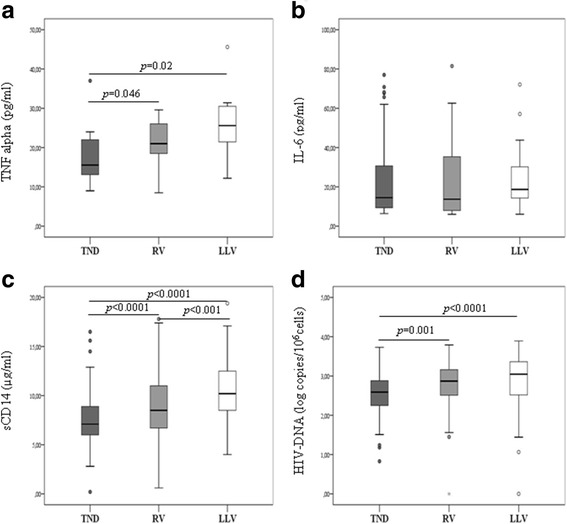
Inflammatory markers and HIV-DNA levels in patients with different VL patterns. TNF-α (**a**), IL-6 (**b**), sCD14 (**c**) and intracellular HIV-DNA (**d**) based on plasma VL pattern. TND (Target Not Detected): patients with a sustained undetectable viremia throughout follow-up; RV (Residual Viremia): patients who had at least three detectable VL values but below the threshold value; LLV (Low Level Viremia): patients with at least two VL values over the threshold value but below 200 copies/ml

Biomarkers of immune activation, TNF-α and IL-6 were measured in participant’s plasma at the end of follow-up. There was no difference in the proportion of patients with TNF-α values above LOD among groups (TND and RV group: 12.4%; LLV group: 7.1%; *p* = 0.196). However, median plasma levels of TNF-α were significantly higher in the patients with RV [23 pg/ml (IQR 18.2–26.2 pg/ml)] or LLV [25.6 pg/ml (IQR 22.7–30.1 pg/ml)] than in the group with undetectable VL [15.5 pg/ml (IQR 13.1–21.0 pg/ml)] (TND vs RV, *p* = 0.046; TND vs LLV, *p* = 0.02) (Fig. 1a).

Interestingly, more patients in the TND group had IL-6 levels above LOD when compared with the LLV group (44.2% vs 24.8%; *p* < 0.0001), nevertheless the median IL-6 levels were slightly lower in the TND group than in the LLV group, even if the difference was not statically significant (TND: 14.5 pg/ml [IQR, 9.3–30.7]; LLV: 20.3 pg/ml [IQR, 14.3–33.1; *p* = 0.48) (Fig. 1b). A significant correlation between IL-6 and TNF-α levels (*r* = 0.50; *p* = 0.015) was found when the parameters were detectable (23 out 321) (data not shown).

### Marker of microbial translocation

To assess microbial translocation, the soluble form of the LPS coreceptor CD14 (sCD14) was measured. Ninety-seven percent of patients showed detectable levels of sCD14. Significantly lower levels of sCD14 were detected in the TND group (7.1 μg/ml [IQR, 6–8.9]) and RV group (8.7 μg/ml [IQR, 6.8–11]) compared the LLV group (10 μg/ml [IQR, 9–12.5], and a significant difference was detected between the TND and RV groups (Fig. 1c). This difference persisted among groups after adjusting for potential confounders such as gender, type of antiretroviral regimen, cART duration, stage of disease, and years since diagnosis (Table 2).

**Table 2:** Independent variables associated with viral load pattern (TND, RV, LLV)

Variables	OR^a^	95% CI	pV
*Gender*			
Male	Ref.	−	−
Female	1.22	0.57–2.61	0.614
*Age* (years, continuous)	0.98	0.94–1.02	0.377
*Therapy regimen*			
INI	Ref.	−	−
NRTIs + NNRTIs	0.11	0.03–0.43	0.001
NRTIs + PIs	0.26	0.08–0.88	0.030
Others	0.22	0.05–0.95	0.043
*Duration of therapy* (years, continuous)	1.08	0.94–1.23	0.267
*Stage of disease*			
A1-A3	Ref.	−	−
B1-B3	0.87	0.27–2.81	0.818
C1-C3	1.07	0.48–2.35	0.872
*Years from testing* (years, continuous)	0.96	0.85–1.08	0.494
*HBV*			
Negative	Ref.	−	−
Positive	2.25	0.67–7.59	0.191
*HCV*			
Negative	Ref.	−	−
Positive	0.77	0.23–2.54	0.664
*sCD14* (continuous)	1.66	1.32–2.08	<0.001
*IL-6* (continuous)	0.99	0.96–1.02	0.386
*TNF-α* (continuous)	1.03	0.95–1.10	0.519
*HIV-DNA* (continuous)	1.01	1.00–1.02	0.003

Abbreviations: *INI* integrase inhibitors, *NRTIs* nucleoside reverse transcriptase inhibitors, *NNRTIs* non-nucleoside reverse transcriptase inhibitors, *PIs* protease inhibitors, *HCV* hepatitis C virus, *HBV* Hepatitis B virus, *sCD14* soluble CD14, *IL-6* interleukin- 6, *TNF-α* tumor necrosis factor α

^a^Proportional odds ratio. For example, concerning the variable “gender”, this is the proportional odds ratio of comparing females to males on viral load pattern level given that the other variables are held constant. For females, the odds of LLV vs. combined RV and TND are 1.22 times greater than for males. Likewise, the odds of the combined categories of LLV and RV vs. TND is 1.22 times greater for females compared to males, given the other variables are held constant in the model

### HIV-DNA, VL pattern and inflammatory biomarkers

The association between total HIV-DNA and different degrees of viremia was investigated. Total HIV-DNA was quantified in 90% of samples. When the HIV-DNA levels were stratified on the basis of viremia, a significant difference among groups was observed. Specifically, HIV-DNA load in PBMCs was higher in infected individuals with LLV and RV than in patients with undetectable HIV-RNA levels (LLV group: 3.05 log copies HIV-DNA /10^6^ PBMC [3.0–3.36] vs TND group: 2.59 log copies HIV-DNA /10^6^ PBMC [2.25–2.88], *p* < 0.0001**;** RV group: 2.87 log copies HIV-DNA /10^6^ PBMC [2.53–3.18] vs TND, *p* = 0.001) (Fig. 1d).

The association between HIV-DNA and residual viremia was confirmed by logistic regression analysis (*p* = 0.003) adjusting for age, sex, type of antiretroviral regimen, cART duration, stage of disease, levels of TNF-α, IL-6, sCD14, years since diagnosis, and risk of virological failure. To verify whether the size of the HIV reservoir was associated with different levels of inflammatory biomarkers (IL-6, TNF-α and sCD14) regardless of viremia, a correlation analysis performed on the overall population disclosed no correlation between the size of blood HIV reservoir and the biomarkers analyzed (data not shown).

### Logistic regression analysis

Ordinal logistic analysis showed that two independent variables were significantly associated with VL pattern: sCD14 (OR 1.66, 95% CI 1.32–2.08; *p* < 0.001); HIV-DNA (OR 1.01, 95% CI 1.00–1.02; *p* = 0.003); In addition NRTIs + NNRTIs and NRTIs + PIs were negatively associated to VL pattern compared to INI-containing regimen (Table 2).

## Discussion

This study investigated the association between inflammatory and virological markers in HIV-infected patients responding to therapy but with different VL patterns. To our knowledge, this is one of the few studies to investigate the relation between the size of the HIV reservoir, VL and markers of inflammation in the same population. The main findings were: 1. the link between viremia and sCD14 levels; 2. the association between viremia and HIV-DNA levels; 3. no link between HIV-DNA and markers of inflammation; 4. no link between HIV-RNA and IL-6 levels.

Our data in a large patient sample confirmed the link between residual viremia and the size of viral reservoirs [21–23]. However, whether residual replication is a cause rather than an effect of higher HIV-DNA levels remains to be established. All patients with sustained undetectable viremia throughout the follow-up showed detectable levels of HIV-DNA, strongly supporting the hypothesis that most HIV-DNA is integrated in virologically controlled patients. Because of the limited quantity of blood obtained from patients, we could not perform assays aimed to determine the frequency of cells carrying replication competent virus. Therefore we cannot exclude the possibility that intracellular HIV-DNA in patients with maximum viral suppression is mainly represented by defective latent virus.

We also observed that TNF-α and sCD14 levels were lower in patients with undetectable viremia than in individuals with residual/low level viremia. Previous findings reported that sCD14 plasma levels in HIV-positive patients were significantly higher than those in HIV-negative subjects but were similar among HIV-positive patients stratified according to plasma VL [25, 26]. A significant difference from our study is that our population was grouped according to their VL patterns. Patients showing lower sCD14 levels had undetectable viremia for at least 48 months and were compared with patients who experienced either at least three low but detectable levels of HIV RNA or at least two in the range of 37–200 copies/mL. Therefore, a lack of maximal viral suppression during follow-up seems to be associated with higher level of sCD14. It is worth noting that although patients with undetectable viremia had lower levels of sCD14 than patients with residual/low level viremia, all individuals showed detectable levels of this MT marker. The lack of a control group in our study precluded a comparison of results with data from healthy donors. However, the normal range of sCD14 was given by the manufacturer and was lower than the range detected in the TND group. This supports previous findings [26, 27] suggesting that residual viral replication contributes to further increase the sCD14 level, but other mechanisms such as a persistent alterations of monocytes-macrophage function and a continuous microbial translocation favored by persistent GALT-associated damage could maintain elevated sCD14 levels in virologically suppressed patients [28, 29].

In our population, lower levels of TNF-α were also detected in the TND group compared to patients with RV and LLV. Although the difference among groups was significantly different, the statistical power of this finding is undermined by the low proportion of patients with biomarker levels above the lower limit of detection. Indeed, one of the limitations of our study is that we did not use a high-sensitivity assay to measure inflammatory markers, so the lowest levels of TNF-α were excluded by the detection limit of the assay.

The lack of correlation between LLV and IL-6 is in agreement with other studies reporting a direct correlation between IL-6 levels and VL values above 400 copies/ml, suggesting that VL drives IL-6 production only at high loads [30, 31].

The lack of association between IL-6 and viremia is also consistent with findings previously reported by Chun et al. who found that low levels viremia were not statistically associated with IL-6 [21]. On the other hand, Bastard and coworkers found an association between HIV VL and IL-6, reporting that low-range IL-6 levels correlated with low-range VL [12]. As explained above, the low range of IL-6 in our study was excluded by the detection limit and only high values of IL-6 were detected. Despite this limitation, our data suggest that high IL-6 levels are not affected by low levels of viremia. We also observed a higher percentage of patients with IL-6 levels above the LOD in the TND group than in the others. This finding was somewhat unexpected, but several factors other than the virus may contribute to the inflammatory state seen during HIV infection. [32]. Indeed high IL-6 levels were associated with higher body mass index, smoking, comorbidities, renal function and serum lipid levels, suggesting multiple determinants of inflammation and/or IL-6 production during HIV infection. Our data support these findings, since the HIV subjects enrolled in this study had been infected for a long time, had a long therapeutic history and most of them showed other comorbidities, such as coinfections, cardiovascular diseases, renal disease or tumors.

We also evaluated the possibility that HIV-DNA levels could be correlated with inflammatory markers such as sCD14 and IL-6, but no correlation was found between the size of the blood reservoir and sCD14 or IL-6. This data is in agreement with other findings reporting no association between intracellular HIV-DNA levels and inflammatory biomarkers such as IL-6, C-reaction protein, soluble TNF receptor I and immune activation markers on CD4+ and CD8 + T cells [21, 24] in ART-treated patients. Interestingly, a strong positive correlation between HIV-DNA and CD8+ T cell activation was found in viremic patients with primary or chronic infection, whereas there was no correlation between T-cell activation and HIV-DNA in patients with successfully treated chronic infection [33]. In this framework, our data confirm that inflammation is not directly associated with the size of the HIV reservoir and emphasize the role of other factors in maintaining elevated levels of immune activation in ART-treated subjects.

Finally and unexpectedly, we observed that patients with low level viremia were more likely to be treated with INIs. Indeed, about half of patients with LLV, treated with a cART containing INI, were treated with a therapy combined with PIs, therefore we cannot rule out the possibility that the above association could be mainly due to the simultaneous usage of both drugs. However, since the remaining patients were treated with INIs combined with different drugs we believe that this result may be due to a selection bias, since patients starting therapeutic regimen containing integrase inhibitor, were mostly represented by patients with more previous treatment failures and with a history of non-adherence to ART. The same consideration could explain, at least in part, the discrepancy of our finding with the literature data reporting that PIs containing regimens increase the risk to have LLV [34, 35].

The study has some limitations. We evaluated only three biomarkers and hence cannot rule out the possibility that other inflammatory parameters might have provided useful information. However we chose three markers associated with disease progression and reported to be predictors of clinical events and mortality during HIV infection [14, 36–40]. Our analysis did not have multiple longitudinal measures of inflammatory markers or HIV-DNA, so we could not investigate the change in these markers over time or their relation with the VL value.

## Conclusion

In summary, our results confirm that a full viral suppression is associated with both a smaller HIV reservoir and lower sCD14 levels, emphasizing the need to maintain VL at undetectable levels. However the lack of normalization of inflammatory markers in patients with persistent undetectable viremia and the fact that HIV-DNA load was not associated with inflammation strongly suggests that HIV infection triggers inflammation, but other mechanisms play a major role in maintaining the inflammatory status over time.

## Abbreviations

ART: antiretroviral therapy
cART: combination ART
IL-6: interleukin-6
INIs: integrase inhibitors
LLV: low level viremia
MT: microbial translocation
NNRTIs: non-nucleoside reverse transcriptase inhibitors
NRTIs: nucleoside reverse transcriptase inhibitors
PIs: protease inhibitors
RV: residual viremia
TND: target not detected
TNF-α: tumor necrosis factor alpha
VL: Viral Load
